# Individual Sensory Modality Dominance as an Influential Factor in the Prefrontal Neurofeedback Training for Spatial Processing: A Functional Near-Infrared Spectroscopy Study

**DOI:** 10.3389/fnsys.2022.774475

**Published:** 2022-02-10

**Authors:** Takeshi Sakurada, Mayuko Matsumoto, Shin-ichiroh Yamamoto

**Affiliations:** ^1^Department of Robotics, College of Science and Engineering, Ritsumeikan University, Shiga, Japan; ^2^Functional Brain Science Laboratory, Center for Development of Advanced Medical Technology, Jichi Medical University, Tochigi, Japan; ^3^Department of Neurosurgery, Jichi Medical University, Tochigi, Japan; ^4^Graduate School of Systems Engineering and Science, Shibaura Institute of Technology, Saitama, Japan

**Keywords:** neurofeedback, functional near-infrared spectroscopy, working memory, prefrontal cortex, individual differences, sensory modality

## Abstract

Neurofeedback is a neuromodulation technique used to improve brain function by self-regulating brain activity. However, the efficacy of neurofeedback training varies widely between individuals, and some participants fail to self-regulate brain activity. To overcome intersubject variation in neurofeedback training efficacy, it is critical to identify the factors that influence this type of neuromodulation. In this study, we considered that individual differences in cognitive ability may influence neurofeedback training efficacy and aimed to clarify the effect of individual working memory (WM) abilities, as characterized by sensory modality dominance, on neurofeedback training efficacy in healthy young adults. In particular, we focused on the abilities of individuals to retain internal (tactile or somatosensory) or external (visual) body information in their WM. Forty participants performed functional near-infrared spectroscopy-based neurofeedback training aimed at producing efficient and lower-level activity in the bilateral dorsolateral prefrontal cortex and frontopolar cortex. We carried out a randomized, sham-controlled, double-blind study that compared WM ability before and after neurofeedback training. Individual WM ability was quantified using a target searching task that required the participants to retain spatial information presented as vibrotactile or visual stimuli. Participants who received feedback information based on their own prefrontal activity showed gradually decreasing activity in the right prefrontal area during the neurofeedback training and demonstrated superior WM ability during the target searching task with vibrotactile stimuli compared with the participants who performed dummy neurofeedback training. In comparison, left prefrontal activity was not influenced by the neurofeedback training. Furthermore, the efficacy of neurofeedback training (i.e., lower right prefrontal activity and better searching task performance) was higher in participants who exhibited tactile dominance rather than visual dominance in their WM. These findings indicate that sensory modality dominance in WM may be an influential neurophysiological factor in determining the efficacy of neurofeedback training. These results may be useful in the development of neurofeedback training protocols tailored to individual needs.

## Introduction

Neurofeedback is a neuromodulation technique that aims to self-regulate brain activity patterns to improve specific functions (Ehlis et al., 2018; Xiang et al., 2018; Jeunet et al., 2019). In healthy individuals, theta electroencephalography (EEG) power increases during neurofeedback training have been shown to assist in improving motor sequential learning (Rozengurt et al., 2016), and high sensorimotor rhythm power has been associated with better motor performance in a sporting task (Cheng et al., 2015). Moreover, neurofeedback training has been noted as a useful protocol for improving cognitive functions such as attention (Wang and Hsieh, 2013), working memory (WM) (Escolano et al., 2011) and mental rotation (Hanslmayr et al., 2005). In clinical fields, neuromodulation by neurofeedback has been used successfully to alter brain activity in a wide range of disorders, such as attention deficit hyperactivity disorder (Leins et al., 2007) and Parkinson’s disease (Subramanian et al., 2011).

Although EEG and fMRI are the imaging techniques most commonly used for neurofeedback training, the use of functional near-infrared spectroscopy (fNIRS) has increased over the last 10 years (Ehlis et al., 2018; Kohl et al., 2020). fNIRS recording has several advantages. For example, if the probes are in close contact with the head, we can measure brain activity, regardless of posture or any slight body movement. Furthermore, fNIRS is resistant to electrical noise, an especially useful feature when conducting neuroimaging experiments. For instance, using fNIRS-based neurofeedback, self-regulation of the right dorsolateral prefrontal cortex (DLPFC) has been shown to contribute to improved emotional regulation (Yu et al., 2021), and postural stability has been found to be affected by greater supplementary motor area activity (Fujimoto et al., 2017). Moreover, neurofeedback of the premotor area during a rehabilitation protocol requiring motor imagery of a paretic hand’s movements enhanced the recovery of finger motor function in patients with hemiplegic stroke. Specifically, the motor imagery-related premotor activity changes during neurofeedback training significantly correlated with functional recovery (Mihara et al., 2013). Altogether, these reports demonstrate that fNIRS-based neurofeedback might constitute an effective approach to neuromodulation. However, it is worth noting that acquiring higher brain activity does not always correlate with improvement in a specific brain function. Lower (i.e., efficient) brain activity can also lead to high brain function performance (Jansma et al., 2001; Ramsey et al., 2004; Koike et al., 2013). Therefore, the direction of self-regulation of brain activity must be appropriately determined in neurofeedback training.

Although various types of neurofeedback training have been reported to be useful for brain functional training, the efficacy of neurofeedback training varies widely among individuals, and a certain population obtains no benefit from neurofeedback training and fails to self-regulate brain activity patterns (Alkoby et al., 2018; Diaz Hernandez et al., 2018; Kadosh and Staunton, 2019). A recent review pointed out that participants who failed to self-regulate during neurofeedback training represented about 50% of the population (Alkoby et al., 2018). For instance, a neurofeedback training protocol that aimed to improve WM ability by increasing upper alpha power indicated that three of nine participants failed to control their alpha power after several training sessions (Escolano et al., 2011). Moreover, during neurofeedback training to modulate sensorimotor rhythm, approximately half of the participants did not learn to regulate their own brain activities even after numerous training sessions (Weber et al., 2011).

To overcome these individual differences in the efficacy of neurofeedback training, we need to identify the predictor (influential factor) of the neuromodulation. Although the factors influencing neurofeedback training are not fully understood, the issue has been discussed recently, and several factors have been pointed out (Alkoby et al., 2018; Diaz Hernandez et al., 2018; Kadosh and Staunton, 2019). First, the effect of psychological factors such as mental strategy, motivation, and mood has been discussed frequently. The most successful mental strategies during neurofeedback training were found to be related to positive thoughts such as those of friends, love, or family (Nan et al., 2012). Another study demonstrated that the psychological effects depended on the targeted EEG component. Specifically, individuals with no specific mental strategy showed improvements from neurofeedback training for sensorimotor rhythm, and the efficacy of neurofeedback training for gamma power did not vary among individuals, regardless of their mental strategies (Kober et al., 2013). The authors pointed out the disadvantage of overloading cognitive resource data from the use of a specific mental strategy during neurofeedback training. Second, in addition to psychological factors, neurophysiological factors also influence the ability to regulate brain activity using neurofeedback. For instance, alpha powers in the eyes-closed and eyes-open resting state before neurofeedback training were significantly correlated with successful EEG learning (Wan et al., 2014). Another study found that an individual’s ability to modulate sensorimotor rhythm in early neurofeedback training sessions predicted later neurofeedback training efficacy (Weber et al., 2011). Thus, even if the psychological state is similar among trainees, different functional brain characteristics might affect the efficacy of neurofeedback training.

Regarding these neurophysiological factors, we should consider the possibility that individual differences in the qualitative characteristics of cognitive function determine neurofeedback training efficacy. We recently reported a wide variation in the sensory modalities that individuals are good at processing; hereafter, we refer to these individual differences as modality dominance. Specifically, individual cognitive abilities in motor imagery or attention control can be characterized by the modality dominance, and this can lead to better processing of internal body information such as tactile or somatosensory stimuli or external body information such as visual stimuli (Sakurada et al., 2016, 2017, 2019a,b). In these studies, individuals with visual motor imagery dominance showed better motor performance when using an external focus attentional strategy, which required that the participant’s attention was focused on a body movement outcome. Conversely, individuals with kinesthetic motor imagery dominance demonstrated better motor performance when using an internal focus attentional strategy, which required that the participant’s attention was focused on the body movement itself. Furthermore, individual WM ability that relates to spatial information processing (Robertson et al., 2001) can be characterized by modality dominance. When participants were required to retain spatial information, they could be grouped into those who were good at retaining vibrotactile-based or visual-based spatial information (Matsumoto et al., 2020). These findings indicate that modality dominance is one of the important parameters in characterizing the qualitative aspects of cognitive function, but whether distinct qualitative differences in cognitive function among individuals affects neurofeedback training efficacy remains unclear.

This study aimed to examine whether modality dominance in WM influences the efficacy of neurofeedback training. Based on our previous findings regarding the neural basis of individual differences in WM modality dominance (Matsumoto et al., 2020), the neurofeedback protocol used in this study provided feedback of the bilateral DLPFC and frontopolar cortex (FPC), which are also critical for WM (Owen et al., 2005; Pleger et al., 2006; Slotnick and Moo, 2006; Kaas et al., 2007; Giglia et al., 2014). We hypothesized that the individual modality dominance in WM is one of the influential factors in neurofeedback training. Specifically, we predicted that the degree of neuromodulation in the prefrontal area and cognitive performance after neurofeedback training would highlight differences between individuals exhibiting tactile- versus visual dominance.

## Materials and Methods

### Participants

Forty healthy participants (mean age ±SD, 22.4 ± 3.2 years; 19 males, 21 females) were recruited from the student population at Jichi Medical University. All participants were right-handed as assessed by the Edinburgh Handedness Inventory (laterality 96.4 ± 8.9) (Oldfield, 1971). This study was conducted in accordance with the Declaration of Helsinki and approved by the Institutional Review Board at Jichi Medical University. All participants provided written informed consent prior to participation. Each participant completed the following experimental protocol in 1 day, including the fNIRS-based neurofeedback training task that aimed to regulate bilateral prefrontal activities and a behavioral task that was used to evaluate WM ability before and after neurofeedback training.

### Regulation of the Prefrontal Activity by Functional Near-Infrared Spectroscopy-Based Neurofeedback Training

#### Experimental Setup

**FIGURE 1:**
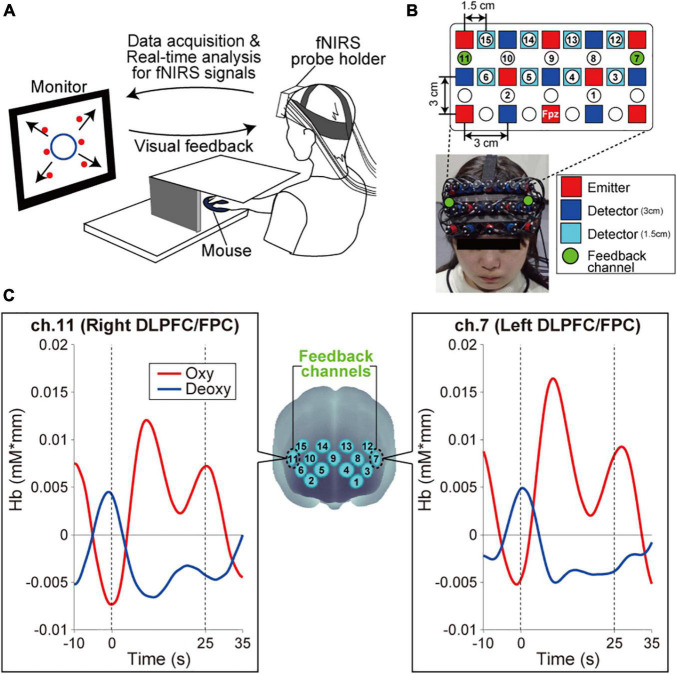
Experimental setup. **(A)** Neurofeedback training set up. The monitor presented visual feedback on the status of participants’ bilateral prefrontal cortex in real-time. Furthermore, the six red circles on the monitor indicated the visual stimuli that participants were required to remember. Specifically, in each task block, the all-red circles were sequentially presented individually in random order at the predetermined fixed positions {with the center of the monitor as the origin, the coordinates of the six circles were [*x*(cm), *y*(cm)] = (–10, 8), (10, 8), (–10, 0), (10, 0), (–10, –8), and (10, –8)}. The appearance order changed with every task block. The participants were required to remember the spatial appearance order of six visual stimuli on the monitor. **(B)** Configuration of the fNIRS probe. Probes were placed over the prefrontal area. The channels, numbered 1–15, indicated the channels that output fNIRS signals that have been removed based on multidistance ICA. Ch.7 and ch.11 conveyed signals from the left and right DLPFC/FPC, respectively, as neurofeedback. **(C)** Spatial registration of the fNIRS maps onto MNI coordinate space. The left and right panels show typical profiles of oxy-Hb and deoxy-Hb in the feedback channels. Time zero indicates the onset of the task block. After starting the task block, the oxy-Hb signal showed a stronger response than the deoxy-Hb signal.

Each participant sat on a chair facing an LCD monitor (size: H30.5 × W37.7 cm) that presented the visual stimulus and was asked to hold a computer mouse in their right hand. All visual stimuli presented on the monitor were programmed in MATLAB (MathWorks, Inc., Natick, MA, United States). During the fNIRS-based neurofeedback training task, the right hand was hidden by a small rack (Figure 1A). A multichannel fNIRS system (ETG-7100, Hitachi Medical Corporation, Kashiwa, Japan) with probes arranged to cover the prefrontal area was used to measure prefrontal activity. All of the fNIRS channel inputs were sampled at 10 Hz. A 3 × 9 multichannel probe holder contained eight laser sources that emitted at 695 and 830 nm and seven detecting probes that were alternately arranged with an interprobe distance of 3 cm. The midpoint between an emitter/detector pair was defined as the location of a recording channel (the probe set initially had 22 recording channels). Importantly, fNIRS signals reflect changes in hemoglobin that originate in cortical tissue due to brain activation and skin blood flow. To eliminate the impact of skin blood flow on the fNIRS signals, we set eight additional short detecting probes at an interprobe distance of 1.5 cm and applied multidistance independent component analysis (ICA) to the fNIRS analysis (Hirosaka et al., 2004; Morren et al., 2004; Akgül et al., 2006; Kohno et al., 2007; Funane et al., 2014). Signals from recording channels with a 1.5 cm interprobe distance primarily included skin blood flow signals in shallow tissues. Based on these signals, we discriminated between the effects of cortical tissue and skin blood flow on the fNIRS signals. As it was possible to apply multidistance ICA only to the recording channels around the short detecting probes, the number of available recording channels was reduced to 15 after applying multidistance ICA as shown in Figure 1B. All results presented in this study were recorded after multidistance ICA processing from the fNIRS system (i.e., the effects of skin blood flow have been removed based on fNIRS signals from the 1.5-cm interprobe channels). The probe holder was placed on the scalp with the lowest-row center emitter located at the participant’s Fpz position according to the standard international 10–20 system (Figure 1B). To spatially register fNIRS maps onto the Montreal Neurological Institute coordinate space, we individually measured scalp landmarks and all fNIRS recording channel positions using a 3D magnetic space digitizer (FASTRAK, Polhemus, United States). We then used the position data from all of the participants’ recording channels to estimate spatial profiling without MRI (Singh et al., 2005). The estimated spatial profiling of each recording channel is shown in Table 1.

**TABLE 1:** Spatial profiling of each recording channel.

Ch.	Localization	Brodmann area	Probability
1	Left FPC	10	1
2	Right FPC	10	1
3	Left FPC	10	0.96
	Left DLPFC	46	0.04
4	Left FPC	10	1
5	Right FPC	10	1
6	Right FPC	10	0.99
	Right DLPFC	46	0.01
7	Left DLPFC	46	0.82
	Left FPC	10	0.18
8	Left FPC	10	0.84
	Left DLPFC	9	0.16
9	Right FPC	10	0.86
	Right DLPFC	9	0.14
10	Right FPC	10	0.80
	Right DLPFC	9	0.20
11	Right DLPFC	46	0.77
	Right FPC	10	0.23
12	Left DLPFC	9	0.70
	Left DLPFC	46	0.24
	Left FPC	10	0.05
	Left includes FEF	8	0.01
13	Left DLPFC	9	0.82
	Left includes FEF	8	0.18
14	Right DLPFC	9	0.81
	Right includes FEF	8	0.19
15	Right DLPFC	9	0.84
	Right DLPFC	46	0.06
	Right FPC	10	0.05
	Right includes FEF	8	0.05

*FPC, frontopolar cortex; DLPFC, dorsolateral prefrontal cortex; FEF, frontal eye fields.*

#### Procedure

The neurofeedback training task consisted of eight sessions, each comprising seven alternating 15-s rest and 25-s task blocks with an additional rest block inserted at the end of each session (i.e., 15 blocks per session).

During the task blocks, the monitor reported neurofeedback information as a circle in which the diameter reflected the online prefrontal activities in channels 7 and 11, corresponding mainly to the bilateral DLPFC and FPC. Although we measured oxygenated and deoxygenated hemoglobin (oxy- and deoxy-Hb), we used the oxy-Hb signals to calculate the neurofeedback information because of their greater sensitivity to changes in cerebral blood flow and higher signal-to-noise ratio than deoxy-Hb signals (Toronov et al., 2001; Strangman et al., 2002). As shown in Figure 1C, we also confirmed that the oxy-Hb signal change was stronger than that of deoxy-Hb in this study. Thus, the circle diameter was determined based on the oxy-Hb signals of ch.7 and ch.11. Furthermore, as the fNIRS signals are relative values, we should avoid using the values directly. Instead, the online oxy-Hb signals during the task blocks in each feedback channel were normalized to the mean and standard deviation during the 10 s prior to each task block (i.e., z-scoring). We then averaged *z*-score values between channels 7 and 11. The circle size increased when the averaged *z*-score among the two channels decreased. The circle was the largest (diameter: 45 cm) when the brain activity in the task block was below the average activity level for 10 s before starting the task block. Under this neurofeedback setting, participants were instructed to make the circle on the monitor as large as possible. In other words, the current neurofeedback training protocol aimed to acquire more efficient (i.e., lower) activities in the bilateral DLPFC/FPC during spatial WM processing. Regarding task instructions related to brain activity, we only explained that the circle enlarged in response to cognitive brain activity for properly holding the target spatial information. We did not explicitly instruct the desired brain activity change (i.e., whether the participants should increase or decrease brain activity). This neurofeedback setting was based on the previous reports that decreasing prefrontal activity is associated with familiarity or a higher skill level during cognitive tasks (Jansma et al., 2001; Ramsey et al., 2004; Koike et al., 2013).

To drive spatial WM processing in the prefrontal area, we set up a cognitive-motor task during the task blocks. For the cognitive-motor task, the monitor sequentially showed six small visual stimuli. Each visual stimulus shown was a red circle with a diameter of 1 cm. The six visual stimuli were sequentially and individually presented in random order at predetermined fixed positions (top-left, top-right, middle-left, middle-right, bottom-left, or bottom-right on the monitor). A series of visual stimuli was presented just after the start of each task block, and the order was different for every task block. The participants were given information on the six visual stimuli (number, position, and randomness of appearance order). They were then instructed to remember the sequential patterns (spatial appearance order) of the six stimuli and to verbally answer the order of the visual stimuli in the rest block immediately after each task block. The participants were also required to press the computer mouse button at about 1 Hz with their index fingers. We utilized this cognitive-motor task during the task blocks, because the effect of cognitive processing cannot be assessed when a task is too easy for participants (Landers et al., 2005; Wulf et al., 2007, 2009) and individual differences in cognitive ability may not appear when the task is of insufficient difficulty (Sakurada et al., 2017).

We randomly assigned the participants to Real or Sham groups (each group contained 20 participants), but the group assignment for each participant was not provided to the experimental operator or the participant. The circle diameter for the Real group was determined in real-time based on each participant’s own oxy-Hb signals in ch.7 and ch.11. Conversely, the circle size for the Sham group was based on the prerecorded oxy-Hb signals of another person and was unrelated to the participant’s own cortical activation.

#### Functional Near-Infrared Spectroscopy Offline Analysis

##### Preprocessing

After applying the multidistance ICA, to remove baseline drift, the individual time course data from the oxy-Hb signals from each channel were high-pass filtered using a cut-off frequency of 0.0125 Hz. Next, to remove blocks containing motion-related artifacts, we applied an artifact detection algorithm based on HOMER2 software [MGH-Martinos Center for Biomedical Imaging (NITRC, 2021: Homer2: Tool/Resource Info)]. As no blocks containing artifacts were detected, we analyzed the entire oxy-Hb time course data obtained from this study.

##### General Linear Model Analysis

General linear model analysis (Friston et al., 1994a,b) can be used to detect task-related hemodynamic changes in the cortex from fNIRS data (Schroeter et al., 2004; Plichta et al., 2007). To identify neuromodulation in the prefrontal regions related to the WM processing of spatial information, we used GLM analysis with least-squares estimation of the oxy-Hb signals. For the preprocessed oxy-Hb signals, a Gaussian function with a peak time of 6 s and full width half maximum of 5.4 s was used as a hemodynamic response function to better mimic brain signals. The resulting beta values at each recording channel estimated by the GLM analysis were then used in the group analysis to evaluate the degree of neuromodulation during the neurofeedback training.

As described previously, the participants were instructed to enlarge the circle size on the monitor. This means that the participants needed to reduce the average activity of the neurofeedback channels (7 and 11). However, it should be noted that there were some potential patterns of brain activity in which participants were considered to have achieved the neurofeedback training goal. Specifically, the participants could enlarge the circle size not only by a simultaneous decreasing of both the left and right neurofeedback channel activities but also by a large decrease of either the left or right activity. Therefore, as channels 7 and 11 might show different activity changes during the neurofeedback training, we independently analyzed the activity changes in each channel, rather than the average activity between the two.

#### Statistical Analysis

The beta values from all of the recording channels were analyzed by three-way repeated-measures analysis of variance (ANOVA) with session (1st or 8th session) and feedback channel (ch.7 or ch.11) as within-subject factors and neurofeedback training group (Real or Sham group) as a between-subject factor. Regarding the effect size, we applied a partial eta squared, which is robust for the number of factors. To evaluate the degree of neural activity change in the feedback channels, the beta values in the first session were compared with those in the eighth session by a *post hoc* test (simple-simple main effect test).

### Target Searching Task for Evaluating Spatial Working Memory Ability

#### Experimental Setup

Based on our previous study (Matsumoto et al., 2020), we applied a target searching task to quantify individual WM modality dominance during spatial processing. The participants performed this searching task in a booth different from that used in the neurofeedback training task. Each participant was seated on a chair and asked to hold a digitizing-pen on a drawing tablet (Intuos4 PTK-1240/K0, Wacom, Japan) with their right hand. An LCD monitor (size: H30.5 × W37.7 cm) used to present the visual stimulus was placed horizontally at 16.5 cm above the tablet. As their right hand was hidden by a cloth and the monitor, the participants could not see it directly during the experimental tasks. Visual stimuli such as task instructions presented on the monitor were programmed in MATLAB using the Cogent Toolbox (University College London, London, United Kingdom). The Cogent Toolbox also recorded the position of the digitizing-pen tip with sampling at 60 Hz. A vibration motor presenting a vibrotactile stimulus was attached to the tip of the index finger on the right hand.

#### Procedure

The searching task required participants to find six targets appearing somewhere in the search area by moving the digitizing-pen on the drawing tablet (search area: H30.5 × W37.7 cm). The targets were located randomly and appeared individually in a predetermined, sequential order. Note that the target locations and appearance orders in this searching task were unrelated to those of the red visual stimuli presented during the neurofeedback training task. We introduced two experimental conditions, a tactile condition and a visual condition, that differed in the sensory modality of the stimulus cues presenting the target locations and orders of appearance. Under the tactile condition, when the tip of the digitizing-pen came into a target area on the tablet (diameter: 10 cm), a vibrotactile stimulus was presented to the right index finger from the vibration motor to indicate the target location. Conversely, under the visual condition, a circular visual cursor was presented on the monitor just above the corresponding digitizing-pen position to indicate the target location. Regarding the target settings, we had prepared different target locations and their appearance orders between the tactile and visual conditions and among the participants. However, the six predetermined target locations and their appearance orders within each participant were fixed throughout the trials under each condition.

In each trial, the participants were first required to move the digitizing-pen to the center of the search area on the tablet. Then, the background color of the monitor changed as a start cue for the participants to begin searching for the first target. When the digitizing-pen entered a target area, the vibrotactile or visual stimulus was presented, and the sensory stimuli continued until the tip of the digitizing-pen moved out of the target area. If the digitizing-pen remained in the target area for 0.7 s, a beep signal informed the participant of successful target detection. Then, the participants immediately began searching for the next target. Finally, each trial finished when the participant found all six targets. Participants were also instructed to find all six targets as quickly as possible. Therefore, they had to retain spatial information, namely, the target locations and orders of appearance, in the repeated trials. We expected that participants would gradually show efficient searching as a learning effect if they could retain the spatial information of the target under each condition.

Before the neurofeedback training task, the participants performed alternating the tactile and visual conditions a total of 20 trials as successive trials, so 10 trials were performed in each condition (the Pre-WM task). The first trial was randomly assigned as the tactile or visual condition for each participant. As described previously, the target locations for each participant differed between the tactile and visual conditions. Therefore, we can expect no transfer of spatial information from the tactile condition to the visual condition and vice versa. Furthermore, to align the task difficulty between the two conditions, the total distance between all targets (i.e., the sum of the straight-line distances connecting the six targets) was the same for both conditions. The participants were required to hold the two patterns of target spatial information from both conditions simultaneously, so we set the number of trials needed to properly retain the information. In a preliminary experiment, we estimated the number of trials required for the searching cost to finally reach the plateau. After the neurofeedback training task, the participants performed alternating both conditions a total of 20 trials again (the Post-WM task), and the target locations in the Post-WM task were the same as those in the Pre-WM task. Thus, the participants can refer to the target spatial information held during the Pre-WM task to improve searching performance during the Post-WM task. If the current neurofeedback training has a positive effect on individual WM ability, the participants in the Real group would show higher searching performance during the Post-WM task than those in the Sham group.

#### Analysis

To evaluate individual WM ability, we applied the same index as that in our previous study (Matsumoto et al., 2020). Specifically, we calculated the searching cost based on searching time (Time) and normalized the movement distance (Dis) in each trial. The searching time was the duration taken to find all of the targets, and the normalized movement distance was the distance moved by the right hand divided by the shortest distance connecting the six targets with a straight line. We defined the “searching cost” using Eq. (1):


(1) $$SearchingCost_{i}=\sqrt{Time_{i}^{2}+{\left(Dis_{i}-1\right)}^{2}}$$

Where subscript *i* denotes the trial number (1–10 in each condition). The searching cost in each trial indicates the distance from the (0, 1) coordinate on a Time–Dis plane. We can deduce that the searching cost reflects the individual WM ability because retaining spatial information for hidden targets can optimize the searching movement path on the drawing tablet (i.e., participants can search for the targets in a shorter distance and a shorter searching time). In this scenario, lower searching costs indicate a higher WM ability to efficiently retain the target locations and order of appearance. Note that the first trial in the Pre-WM task was excluded from the statistical analysis, because the participants did not know the target locations during the first trial and needed to search randomly for them without relying on their WM. Thus, the searching costs in the 2nd to 10th trials in the Pre-WM task and the 1st to 10th trials in the Post-WM task were assumed to reflect the individual WM ability.

Furthermore, to characterize the modality dominance in the WM as a qualitative aspect of cognition in individuals, we compared the searching costs between the tactile and visual conditions at the 10th trial in the Pre-WM task. We subtracted the searching cost under the tactile condition from that under the visual condition as an index of modality dominance. Therefore, positive and negative values indicated tactile-dominant (TD) and visual-dominant (VD) individuals, respectively.

#### Statistical Analysis

The searching costs in the Pre- and Post-WM tasks were analyzed by three-way repeated-measures ANOVA with trial (2nd or 10th trial for the Pre-WM task and 1st or 10th trial for the Post-WM task) and condition (tactile or visual condition) as within-subject factors and neurofeedback training group (Real or Sham group) as a between-subject factor.

Then, to clarify the relationship between individual learning ability and the efficacy of neurofeedback training, we calculated the Pearson correlation coefficients (r) between the amount of decrease in searching cost from the 2nd to the 10th trial and the beta value change. Specifically, we focused on the effect of the performance improvement in the Pre-WM task on the beta value change and the effect of the beta value change on the performance improvement in the Post-WM task.

Furthermore, to evaluate the influence of individual modality dominance in WM on the efficacy of neurofeedback training, we calculated the Pearson correlation coefficient (r) between the beta value change and searching cost in the final trial under the Post-WM task. Then, we compared the beta value change and searching cost in the Post-WM task between TD and VD individuals in the Real group using Wilcoxon rank sum tests. If the individual modality dominance affected the efficacy of the neurofeedback training, the TD and VD individuals were expected to be distributed as distinct clusters on the plane of the beta value change and searching cost. Therefore, we estimated the decision boundary between TD and VD individuals by linear discriminant analysis based on the beta value change and searching cost. We then compared discriminant function values calculated by linear discriminant analysis between TD and VD individuals using a Wilcoxon rank sum test.

## Results

### Neurofeedback Training Task

#### Prefrontal Activity

**FIGURE 2:**
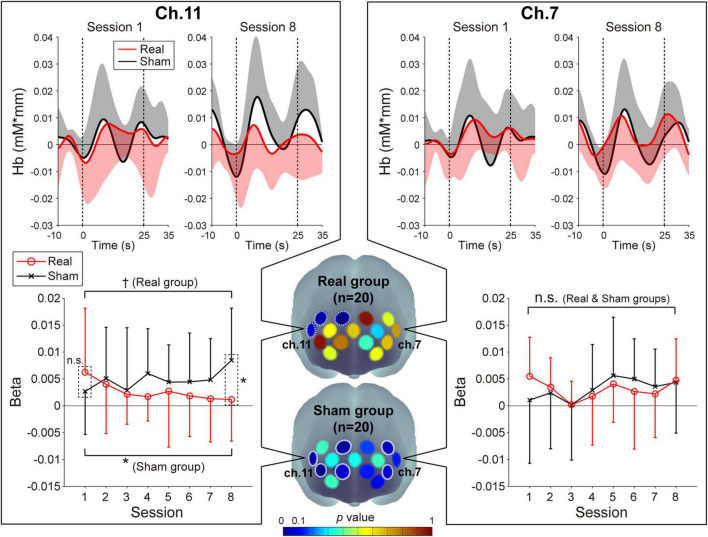
Neuromodulations of the prefrontal cortex activity during the neurofeedback training task. Upper panels: The temporal profiles of the oxy-Hb signals in ch.11 (left panels) and ch.7 (right panels). The red and black lines represent the time courses of the oxy-Hb signals in the Real and Sham groups, respectively. The lighter colored regions around the time course lines denote the standard deviation. The upper or lower directional standard deviation regions are shown for the profiles of the Real and Sham groups, respectively. In ch.11, compared to the first session, the oxy-Hb signal in the final session tended to decrease in the Real group and increase in the Sham group. Lower panels: Beta value transitions in ch.11 (left panels) and ch.7 (right panels). In the first session of ch.11, although there was no significant difference in the beta value between the Real and Sham groups, the Real group showed significantly lower activity than the Sham group at the final session. In contrast, in ch.7, no significant beta value change was observed in both groups. Middle lower 3D brain illustrations: Spatial configurations of the *p*-values from the simple-simple main effect test comparing the beta value in the first session and that in the last session. In the Real group, activities focally decreased in the right hemisphere, including the feedback channel (white dotted circles), while in the Sham group, large activity increases were observed in the bilateral broad region (white solid circles). Error bars denote the standard deviation. ^†^*p* < 0.1, *p < 0.05.

The prefrontal activity patterns in the feedback channels [left prefrontal area (ch.7) and right prefrontal area (ch.11)] and the beta value transitions as estimated by GLM analysis for the oxy-Hb signals are shown in Figure 2. With respect to the beta values of the oxy-Hb signals, we found a marked group-difference in the ch.11 activity patterns. The three-way ANOVA revealed significant interactions for group × session [*F*(1,38) = 7.40, *p* = 0.0098, η_*p*_^2^ = 0.16] and group × channel [*F*(1,38) = 4.82, *p* = 0.034, η_*p*_^2^ = 0.11] and a marginally significant interaction for group × session × channel [*F*(1,38) = 3.13, *p* = 0.085, η_*p*_^2^ = 0.076]. *Post hoc* tests on the Real group revealed that the beta value in ch.11 decreased, which was consistent with the neurofeedback training aims [*p* = 0.057, simple-simple main effect test (1st vs. 8th)], and these decreasing trends were focal and limited to the right hemisphere [ch.14: *p* = 0.0027, ch.15: *p* = 0.029; simple-simple main effect test (1st vs. 8th)]. However, there was no notable beta value change in ch.7 [*p* = 0.73; simple-simple main effect test (1st vs. 8th)]. With respect to the Sham group, individuals showed an increasing trend only in the right feedback channel (ch.11: *p* = 0.031, ch.7: *p* = 0.13; simple-simple main effect). The increasing trends in the Sham group were identified in the broad areas including the left and right hemispheres (ch.3, 5, 6, 11, 12, and 14). The beta value transitions for all of the recording channels are shown in Supplementary Figure 1, and all of the statistical values from the three-way ANOVA of the beta values are shown in Supplementary Table 1.

On the other hand, regarding the beta value of the deoxy-Hb signals, no significant changes were observed compared to the oxy-Hb, as was expected. In both channels, the beta values tended to increase slightly in the Real group and decrease slightly in the Sham group [average beta values: Real group –0.0027 (ch.7, 1st session), –0.0014 (ch.7, 8th session), –0.0024 (ch.11, 1st session), –0.0010 (ch.11, 8th session); Sham group 9.7 × 10^–5^ (ch.7, 1st session), –0.00055 (ch.7, 8th session), –2.8 × 10^–6^ (ch.11, 1st session), –0.0035 (ch.11, 8th session)]. All statistical values calculated from the three-way ANOVA of the beta values are shown in Supplementary Table 2.

#### Cognitive Performance

With respect to the appearance order of the six visual stimuli, all participants exceeded the correct answer rate of 80% (mean ± SD: 96.9 ± 3.9, range: 83.3–100%). No significant correlation was observed between the individual differences in cognitive ability for the current cognitive-motor task and the amount of oxy-Hb beta value change from the 1st to the 8th session (ch.7: *r* = 0.14, *p* = 0.39, ch.11: *r* = 0.22, *p* = 0.16).

### Target Searching Task

#### Searching Cost

**FIGURE 3:**
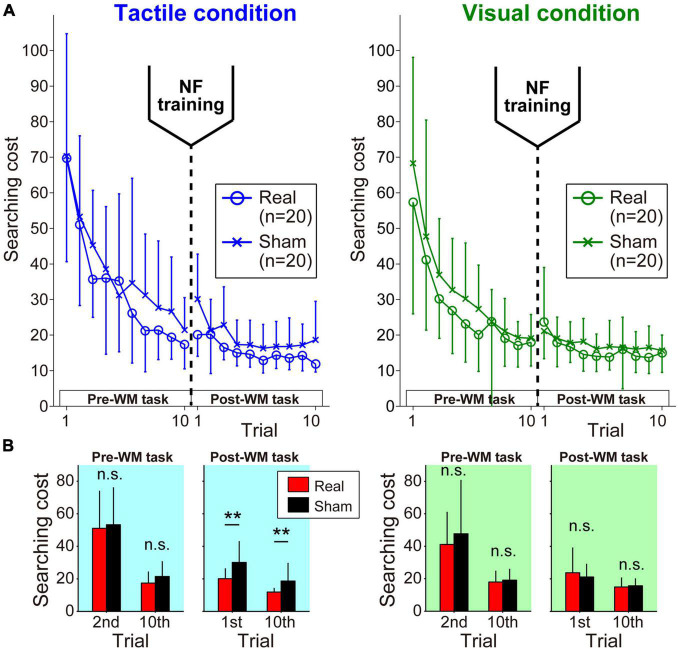
Learning curves during pre- and post-WM tasks. **(A)** In each task phase, the participants successfully reduced the searching cost. **(B)** The Real group showed significantly better performance in the Post-WM task only under the tactile condition. Error bars denote the standard deviation. **p < 0.01.

In the Pre-WM task, all of the participants gradually reduced the searching cost over successive trials. In the Post-WM task involving the same target locations as the Pre-WM task, low searching costs were shown from the first trial, and the participants were able to search more efficiently during repeated trials (Figure 3A).

Here, we focused on the difference in searching costs between the Real and Sham groups (Figure 3B). In the Pre-WM task, a three-way ANOVA of the searching cost showed only a significant main effect of trial [*F*(1,38) = 101.93, *p* = 2.62 × 10^–12^, η_*p*_^2^ = 0.73]. The other factors did not reach the level of statistical significance (*F*s < 2.16, *p*s > 0.15). These statistical results for the Pre-WM task indicated the baseline WM performance before neurofeedback training was not different between the Real and Sham groups. Next, three-way ANOVA for the Post-WM task revealed a significant main effect of trial [*F*(1,38) = 50.36, *p* = 1.82 × 10^–8^, η_*p*_^2^ = 0.57] and a significant two-way interaction of condition × group [*F*(1,38) = 13.43, *p* = 7.52 × 10^–4^, η_*p*_^2^ = 0.26]. The other factors did not reach the level of statistical significance (*F*s < 3.27, *p*s > 0.79). Note that the simple main effect test for the condition × group interaction found that the Real group exhibited significantly lower searching costs than the Sham group under the tactile condition (Tactile condition: *p* = 0.0017, Visual condition: *p* = 0.69). All statistical values of the three-way ANOVA for the searching cost are shown in Supplementary Tables 3, 4.

#### Individual Modality Dominance in Working Memory

Based on differences in the searching cost between the tactile and visual conditions at the 10th trial in the Pre-WM task (i.e., modality dominance in WM), we labeled the 40 participants as TD or VD individuals. Consistent with our previous study (Matsumoto et al., 2020), we also confirmed that the modality dominance in WM ability varied widely among individuals. Specifically, 10 of the participants in the Real group were labeled as TD-individuals and 10 as VD individuals, respectively, whereas eight members of the Sham group were labeled as TD-individuals and 12 as VD individuals, respectively.

### Modality Dominance Dependency in Neurofeedback Training Efficacy

Regarding the tactile condition with significant group difference in the target searching task, neither group showed a significant correlation between the amount of behavioral performance improvement observed in the Pre-WM task (i.e., the amount of decrease in searching cost) and the beta value change (Real group: *r* = –0.01, *p* = 0.96; Sham group: r = 0.29, *p* = 0.22). Similarly, no significant correlation was found between the beta value change and the amount of behavioral performance improvement observed in the Post-WM task (Real group: *r* = –0.32, *p* = 0.16; Sham group: *r* = –0.13, *p* = 0.56).

**FIGURE 4:**
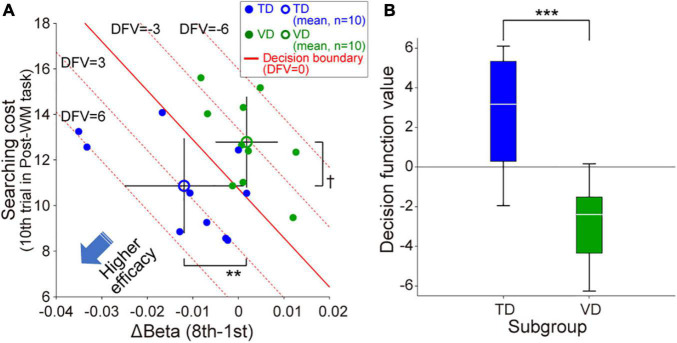
Individual modality dominance dependency of neurofeedback training efficacy in the Real group. **(A)** Scatter plot of the individual neurofeedback training efficacy based on the beta value change in ch.11 and WM task performance in the Post-WM task. TD-individuals are relatively distributed in the lower left of the scatter plot compared with the VD individuals. The red solid line indicates the decision boundary estimated by linear discriminant analysis [i.e., the decision function value (DFV) is zero on this decision boundary]. The red dotted lines represent other DFVs (6, 3, –3, and –6, respectively). Error bars of the mean values denote the standard deviation. **(B)** The decision function values for the TD-individuals were significantly higher than those for the VD individuals. ^†^*p* < 0.1, **p < 0.01, ***p < 0.001.

We then examined whether the neurofeedback training efficacy differed between TD and VD individuals. Figure 4A shows the distribution of the beta value changes in ch.11 during neurofeedback training and WM performance under the tactile condition following the neurofeedback training for the 20 individuals in the Real group. Although there was no significant correlation between the intersubject variance of beta value changes and that of searching costs for the entire cohort (*r* = –0.14, *p* = 0.55), the clusters of TD- and VD individuals seemed to be dissociated. Indeed, the TD-individuals showed significantly greater self-regulation of neural activity than the VD individuals (*p* = 0.0073), and the mean searching cost for TD-individuals was lower than that for VD individuals (*p* = 0.064). Based on the distribution of the Real group, the decision function was estimated as   f  =  –246.4*Δβ –1.1*SC+12.3 (*SC* denotes “searching cost”), and the estimated decision boundary revealed that the TD-individuals were located in the lower left of the scatter plot compared with the VD individuals. Thus, as shown in Figure 4A, when located in the lower left of the plane, the decision function shows a larger value. A larger decision function value indicates higher neurofeedback training efficacy, reflecting greater decreases in right prefrontal activities and better WM performance after neurofeedback training. Based on the decision function value, the neurofeedback training of the TD-individuals was more effective than that of the VD individuals (*p* = 0.00058, Figure 4B). Note that we also confirmed that the Sham group had relatively lower decision function values than the Real group (–10.4 ± 13.1 SD).

## Discussion

The neurofeedback training provided in this study succeeded in lowering neural activity in the right prefrontal area. Moreover, this efficient prefrontal activity facilitated the WM ability to retain internal body information. With respect to the influence of modality dominance on the efficacy of neurofeedback training, the individuals exhibiting tactile dominance showed higher levels of neuromodulation and greater WM performance compared with the individuals with visual dominance. In this study, we focused on the importance of considering individual differences in cognitive function when applying neurofeedback training.

### Effectiveness of Functional Near-Infrared Spectroscopy-Based Neurofeedback Training for Working Memory

The fNIRS-based neurofeedback training used in this study is a useful approach to improving self-regulation of the prefrontal area and facilitating WM performance. In particular, lower prefrontal activity contributed to the ability to retain sensory information. The current level of training efficacy is reasonable given the relationship between lower prefrontal activity and higher cognitive skill (Jansma et al., 2001; Ramsey et al., 2004; Koike et al., 2013). Previously, lower levels of activity in the prefrontal and posterior parietal lobes were shown to be associated with an individual optimal attentional strategy, and this led to a higher motor learning effect (Sakurada et al., 2019b), implying that lower prefrontal activity increases the cognitive resource margin due to higher cognitive processing efficiency. As a result, efficient prefrontal activity may lead to improvements in cognitive or motor performance. Although no direct evidence has supported this interpretation, the lower oxy-Hb signals during the current neurofeedback training may provide a hint of the idea of the promoting cognitive margin. Conversely, a number of previous neurofeedback training strategies have been aimed at increasing the prefrontal activity of the target brain area(s) (Wang and Hsieh, 2013; Hsueh et al., 2016). Several studies have reported that increasing prefrontal activity is an effective approach to improving WM ability in the elderly and in patients with stroke (van Asselen et al., 2006; Jones et al., 2015; Stephens and Berryhill, 2016). These previous studies have demonstrated that increasing neural activity by neurofeedback training is also an effective approach to improving specific brain functions. Increasing activity may improve cognitive performance by maximizing active cognitive resources rather than promoting efficiency in cognitive processing within constant resources. The reason for such a discrepancy that both an increase and a decrease of prefrontal activity can contribute to improving WM ability is unclear. However, age and medical history (especially cerebrovascular disease) may be influential factors for the neuromodulation effect. Therefore, in applying neurofeedback training, it is necessary to make an appropriate choice about increasing or decreasing activity according to the target brain function or training population.

The efficacy of the neurofeedback training presented in this study was limited; the prefrontal neuromodulation contributed to the maintenance of vibrotactile rather than visual information. These results might be due to individual differences in cognitive function as characterized by the ability to process internal body information (Sakurada et al., 2017, 2019a; Matsumoto et al., 2020). Specifically, while most participants have a certain cognitive ability to process visual stimuli, some individuals are less good at processing internal body information such as vibrotactile stimuli. Thus, a greater margin of improvement observed in participants for the tactile condition than for the visual condition might have resulted in significantly better training efficacy for the tactile condition. In addition, note that the protocol included only a short-term training period (i.e., only 1 day), which may have been the reason for the lack of training efficacy seen for the visual stimulation. In a number of previous studies, the neurofeedback training was longer in duration than in the present protocol (Wang and Hsieh, 2013; Hsueh et al., 2016), and longer neurofeedback training might provide greater benefits even for the visual condition. Further investigation is needed on this point.

### Modality Dominance Dependency in Neurofeedback Training Efficacy

Sensory modality dominance in cognitive function is an influential factor in determining the efficacy of neurofeedback training. As in our previous study (Matsumoto et al., 2020), we found large intersubject variability in the modality dominance of WM and demonstrated the relationship between the individual modality dominance and degree of neuromodulation and behavioral performance. Furthermore, as no significant correlation was found between the amount of behavioral performance improvement in the target searching task and the neurofeedback training efficacy, the current findings imply that the individual differences in the amount of neuromodulation during neurofeedback training are not affected by the learning ability. In other words, the sensory modality dominance is a more crucial factor than the individual learning ability, as the individual brain characteristics determine the neurofeedback training efficacy. When not only neurophysiological factors such as alpha power or sensorimotor rhythm (Weber et al., 2011; Wan et al., 2014) but also modality dominance are used to characterize the cognitive traits of an individual, it is possible to predict neurofeedback training efficacy more accurately. Note that we confirmed that 75% of participants in the Real group showed the same sensory modality dominance between Pre- and Post-WM tasks (i.e., tactile or visual dominance was maintained), and the other few participants showed different dominance (tactile changed to visual in one participant and visual changed to tactile in four participants). Therefore, although we found relatively more participants who had a change from visual to tactile, the current neurofeedback training would have no effect on reversing the sensory processing ability between tactile and visual modalities to a specific direction.

With regard to the acquisition of “low prefrontal activity,” which is the purpose of the current neurofeedback training as a successful training efficacy for the self-regulation of brain activity, 90% of individuals with tactile dominance showed decreased prefrontal activity during training sessions. This rate of success was higher than that seen in previous studies (Alkoby et al., 2018). Conversely, the success rate for the neuromodulation of VD individuals was only 30% in terms of the purpose of decreasing prefrontal activity in the current neurofeedback training. Thus, using the statistical approach of comparing Real and Sham groups, which did not consider individual modality dominance, might result in inaccurate predictions of neurofeedback training efficacy for the entire Real group. However, predicting training efficacy based on individual differences in brain function can contribute to optimizing individual training protocols and improving the training success rate.

### Role of the Right Prefrontal Cortex

The efficiency of prefrontal cortex activity was shown to facilitate the WM ability to hold internal body information. Specifically, significant changes in activity were observed during neurofeedback training in the right hemisphere, which corresponds to the DLPFC (Brodmann area 46 and 9) and FPC (Brodmann area 10). The bilateral DLPFC and FPC have been widely recognized as critical structures for WM (Owen et al., 2005). For instance, increasing activity in the right DLPFC with transcranial direct current stimulation led to improved accuracy in memorizing visuospatial locations (Giglia et al., 2014). In addition, the DLPFC and FPC are associated with visual spatial memory (Slotnick and Moo, 2006). Moreover, the left DLPFC and FPC play an important role in processing internal body information such as tactile or somatosensory stimuli. Activity in the left DLPFC correlates with accuracy in discriminating two successive somatosensory stimuli (Pleger et al., 2006). Furthermore, the left FPC is associated with WM representations of haptic information and the integration of spatial and motor components (Kaas et al., 2007). Note that the neurofeedback training protocol used in this study required participants to retain spatial information; therefore, the participants succeeded in self-regulating neural activity in the right prefrontal area that was related to spatial memory. However, we presumed that the modulation of activity in the left DLPFC/FPC failed because the neurofeedback training was based on visual information (i.e., the participants were required to retain spatial information based on visual stimuli). If the neurofeedback training task had required the participants to retain spatial information based on tactile information such as a vibrotactile stimulus, the left DLPFC might also have been successfully modulated. Taken together, the findings suggested that the improvement in the WM ability to hold spatial information with efficient activity levels in the right DLPFC/FPC promoted a behavioral outcome under the tactile condition.

The brain networks between DLPFC and other areas are also important approaches in interpreting the neurofeedback training efficacy. For instance, the prefrontal cortex and posterior parietal cortex are the crucial neural bases for spatial cognition. Persistent activities in these areas reflect not only the maintenance of a WM representation but also the maintenance of a motor intention (Jerde and Curtis, 2013). Furthermore, it has been reported that bilateral primary somatosensory cortices are involved in tactile WM and that DLPFC contributes to bridging the somatosensory cortices from both sides for goal-directed action generation (Zhao et al., 2017). In other words, for the processing WM function, DLPFC forms the frontoparietal network and a network connected with sensory areas. Therefore, the acquisition of efficient prefrontal activity may promote processing in these other connected areas with the prefrontal cortex, leading to a higher adaptive capacity.

### Conclusion

We demonstrate that lowering the activity in the right prefrontal cortex using fNIRS-based neurofeedback training (i.e., improving the efficiency of activity) can facilitate the ability of the WM to retain spatial information. Moreover, individual differences in the sensory modality dominance of the WM, in particular, the ability to hold internal body information, which varies widely among individuals, is an important and newly identified neurophysiological factor that can determine the efficacy of neurofeedback training. Therefore, a customized approach to developing neurofeedback training protocols that are suited to the brain dynamics of the individual will provide more effective neuromodulation methods. Specifically, when applying neurofeedback training to stroke patients with large individual differences in cognitive function, considering the individual sensory modality dominance will provide a tailor-made neurorehabilitation protocol with higher cognitive or motor training effects.

## Data Availability Statement

The raw data supporting the conclusions of this article will be made available by the authors, without undue reservation.

## Ethics Statement

The studies involving human participants were reviewed and approved by the Institutional Review Board at Jichi Medical University. The participants provided their written informed consent to participate in this study. Written informed consent was obtained from the individual(s) for the publication of any identifiable images or data included in this article.

## Author Contributions

TS conceived and designed the experiment, developed the experimental system, wrote the draft of the manuscript, and supervised the study. MM performed the participant experiments. TS and MM analyzed the data. All authors contributed to the discussion of the results, and read and approved the final manuscript.

## Conflict of Interest

The authors declare that the research was conducted in the absence of any commercial or financial relationships that could be construed as a potential conflict of interest.

## Publisher’s Note

All claims expressed in this article are solely those of the authors and do not necessarily represent those of their affiliated organizations, or those of the publisher, the editors and the reviewers. Any product that may be evaluated in this article, or claim that may be made by its manufacturer, is not guaranteed or endorsed by the publisher.
