# Supplementary material for: Individual Sensory Modality Dominance as an Influential Factor in the Prefrontal Neurofeedback Training for Spatial Processing: A Functional Near-Infrared Spectroscopy Study
Source: Front Syst Neurosci. 2022 Feb 10;16:774475. doi: 10.3389/fnsys.2022.774475 (PMC8866872; doi:10.3389/fnsys.2022.774475)
Supplement: Supplementary file 1 [file Data_Sheet_1.pdf]

Supplementary Material

1 Supplementary Figure

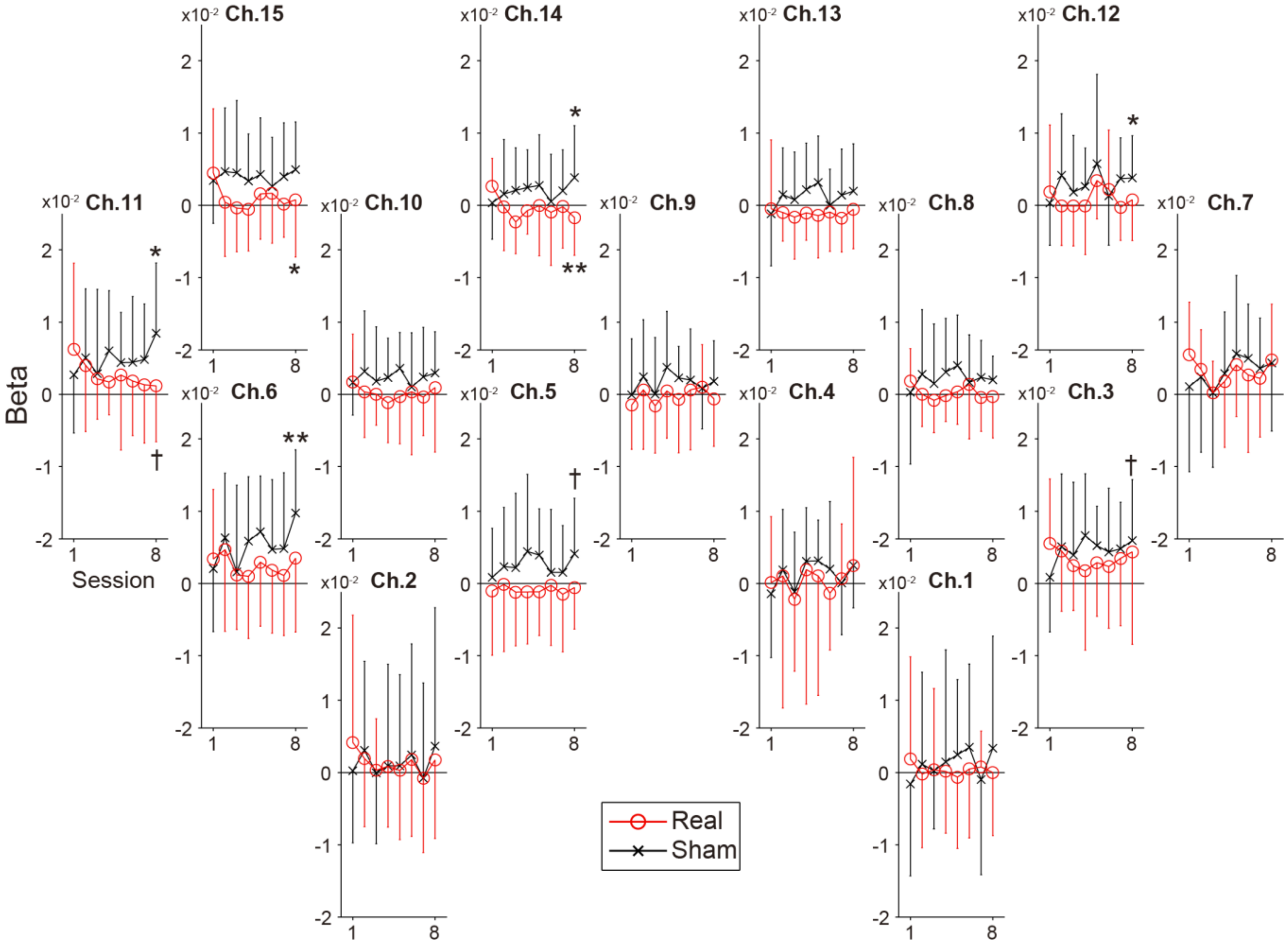

Supplementary Figure 1. Oxy-Hb beta value transitions for all recording channels. The asterisk and dagger signs indicate the significance in the simple-simple main effect test when comparing beta values between the first and eighth sessions in the same group. The error bars denote the standard deviation. †$p < 0.1$, *$p < 0.05$, **$p < 0.01$

2 Supplementary Tables

Supplementary Table 1. Statistical values obtained from the three-way ANOVA of the beta values of oxy-Hb in the first and eighth sessions.

	F-value [*F*(1,38)]	*P*-value	Effect size (Partial η^2)
Group	0.014	0.91	0.00038
Session	0.35	0.56	0.0092
*Group × Session	7.40	0.0098	0.16
Channel	0.54	0.47	0.014
*Group × Channel	4.82	0.034	0.11
Session × Channel	0.23	0.64	0.0059
†Group × Session × Channel	3.13	0.085	0.76

Supplementary Table 2. Statistical values obtained from the three-way ANOVA of the beta values of deoxy-Hb in the first and eighth sessions.

	F-value [*F*(1,38)]	*P*-value	Effect size (Partial η^2)
Group	1.17	0.17	0.030
Session	0.14	0.71	0.0037
†Group × Session	3.61	0.065	0.087
Channel	0.49	0.49	0.013
Group × Channel	1.19	0.28	0.030
Session × Channel	0.93	0.34	0.024
Group × Session × Channel	1.04	0.32	0.027

Supplementary Table 3. Statistical values obtained from the three-way ANOVA of the searching costs in the Pre-WM task.

	F-value [*F*(1,38)]	*P*-value	Effect size (Partial η^2)
Group	1.48	0.23	0.037
*Trial	101.93	2.62×10^{-12}	0.73
Group × Trial	0.093	0.76	0.0024
Condition	2.16	0.15	0.054
Group × Condition	0.016	0.90	0.00041
Trial × Condition	1.38	0.25	0.035
Group × Trial × Condition	0.39	0.54	0.010

Supplementary Table 4. Statistical values obtained from the three-way ANOVA of the searching costs in the Post-WM task.

	F-value [*F*(1,38)]	*P*-value	Effect size (Partial η^2)
†Group	3.27	0.079	0.079
*Trial	50.34	1.82×10^{-8}	0.57
Group × Trial	2.54×10^{-7}	0.99	6.67×10^{-9}
Condition	1.03	0.32	0.026
*Group × Condition	13.43	0.00075	0.26
Trial × Condition	1.94	0.17	0.048
Group × Trial × Condition	2.57	0.12	0.063
